# Functions of the Lyn tyrosine kinase in health and disease

**DOI:** 10.1186/1478-811X-10-21

**Published:** 2012-07-17

**Authors:** Evan Ingley

**Affiliations:** 1 Cell Signalling Group, Laboratory for Cancer Medicine, Western Australian Institute for Medical Research, Centre for Medical Research, The University of Western Australia, Rear 50 Murray Street, Perth, WA, 6000, Australia

**Keywords:** Lyn tyrosine kinase, Src family kinases, Oncoprotein signaling, Immune dysfunction, Leukaemia, Cancer.

## Abstract

**Abstract:**

Src family kinases such as Lyn are important signaling intermediaries, relaying and modulating different inputs to regulate various outputs, such as proliferation, differentiation, apoptosis, migration and metabolism. Intriguingly, Lyn can mediate both positive and negative signaling processes within the same or different cellular contexts. This duality is exemplified by the B-cell defect in *Lyn*^*−/−*^ mice in which Lyn is essential for negative regulation of the B-cell receptor; conversely, B-cells expressing a dominant active mutant of Lyn (*Lyn*^*up/up*^) have elevated activities of positive regulators of the B-cell receptor due to this hyperactive kinase. Lyn has well-established functions in most haematopoietic cells, viz. progenitors via influencing c-kit signaling, through to mature cell receptor/integrin signaling, e.g. erythrocytes, platelets, mast cells and macrophages. Consequently, there is an important role for this kinase in regulating hematopoietic abnormalities. Lyn is an important regulator of autoimmune diseases such as asthma and psoriasis, due to its profound ability to influence immune cell signaling. Lyn has also been found to be important for maintaining the leukemic phenotype of many different liquid cancers including acute myeloid leukaemia (AML), chronic myeloid leukaemia (CML) and B-cell lymphocytic leukaemia (BCLL). Lyn is also expressed in some solid tumors and here too it is establishing itself as a potential therapeutic target for prostate, glioblastoma, colon and more aggressive subtypes of breast cancer.

**Lay Abstract:**

To relay information, a cell uses enzymes that put molecular markers on specific proteins so they interact with other proteins or move to specific parts of the cell to have particular functions. A protein called Lyn is one of these enzymes that regulate information transfer within cells to modulate cell growth, survival and movement. Depending on which type of cell and the source of the information input, Lyn can positively or negatively regulate the information output. This ability of Lyn to be able to both turn on and turn off the relay of information inside cells makes it difficult to fully understand its precise function in each specific circumstance. Lyn has important functions for cells involved in blood development, including different while blood cells as well as red blood cells, and in particular for the immune cells that produce antibodies (B-cells), as exemplified by the major B-cell abnormalities that mice with mutations in the *Lyn* gene display. Certain types of leukaemia and lymphoma appear to have too much Lyn activity that in part causes the characteristics of these diseases, suggesting it may be a good target to develop new anti-leukaemia drugs. Furthermore, some specific types, and even specific subtypes, of solid cancers, e.g. prostate, brain and breast cancer can also have abnormal regulation of Lyn. Consequently, targeting this protein in these cancers could also prove to be beneficial.

## Review

### Lyn as a signaling intermediary

Both receptor and non-receptor protein tyrosine kinases are essential enzymes in many cellular signaling processes regulating cell growth, differentiation, apoptosis, migration, immune responses, adhesion and metabolism [1]. Members of the Src family of tyrosine kinases are signaling intermediates that can control aspects of these and other biological processes [2,3].

**Figure 1:**
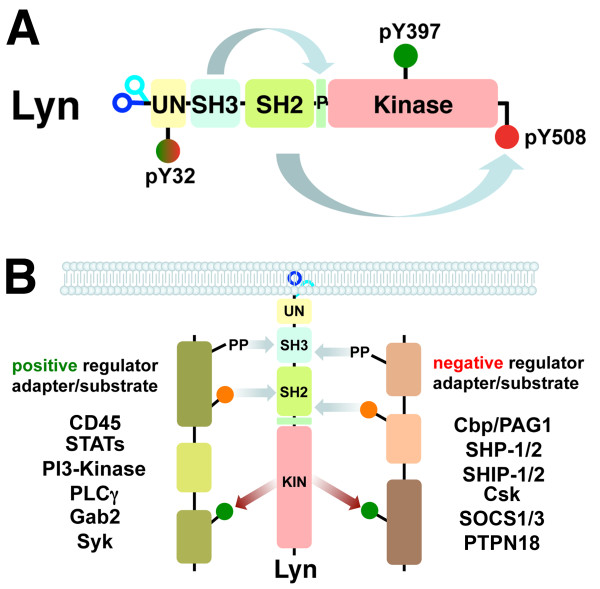
**Regulation of Lyn and Lyn pathways.A**) Domain architecture of Lyn. Schematic of Lyn protein functional domains and motifs, unique (UN), Src Homology 3 (SH3), Src Homology 2 (SH2), and Kinase domains, proline motif in the hinge region (P), amino terminal lipid modifications are indicated; myristoylation (navy blue) and palmitoylation (cyan). Important pY motifs that are phosphorylated in the inactive (pY508, red) and active (pY397, green) kinase are indicated, as is the LynA/p56 isoform-specific motif pY32 that may modulate activity/interactions. Intramolecular interactions between the SH3 domain and the hinge (P) region, as well as the SH2 domain and the C-terminal pY508 motif are shown. **B**) Lyn regulation of positive and negative signaling pathways. Lyn regulates multiple signaling pathways by interacting with and/or phosphorylating different molecules that can mediate both the activation/enhancement as well as the inhibition/termination of signaling networks, as illustrated.

Lyn is a member of the Src family of intracellular membrane-associated tyrosine kinases (SFK). Each member has a unique N-terminal region (SH4) encoding a myristoylation site, and may contain one (e.g. Lyn) or two (e.g. Fyn) palmitoylation sites [4], followed by homologous domains for protein interaction (SH3 and SH2), as well as a kinase (SH1) domain (Figure 1) [5]. Lyn has two splice variants (via exon 2) that result in the generation of p53 and p56 kDa protein isoforms, designated as LynA (p56) and LynB (p53), which differ by a 20 amino acid region in the SH4 domain that encompasses a pY motif (pY32) [6,7]. The reversible N-terminal lipid modification (palmitoylation) and isoform specific pY32 motif potentially complicate understanding Lyn’s function through their latent ability to regulate activity, interactions, and subcellular localization. As with other Src family kinases Lyn is regulated by protein interactions through its SH2/SH3 domains as well as via phosphorylation status (Figure 1A) [8]. In its inactive state Lyn is phosphorylated at its carboxyl terminus by C-terminal Src kinase (Csk) creating a binding site for its own SH2 domain. Lyn’s SH3 domain can bind an intramolecular proline-motif situated between the SH2 and kinase domains (hinge region), helping generate a stabilized inactive kinase confirmation. Activation of Lyn involves dephosphorylation of the C-terminal tyrosine (Y508) by phosphatases such as CD45 [9] and SHP-2 [10], as well as through interactions with SH2 and/or SH3 domain binding motifs, which compete with Lyn's own SH3/SH2 intramolecular interaction sites, thus releasing the auto-inhibitory configuration of the kinase domain. Lyn can then trans-autophosphorylate within the activation loop (Y397) to generate a highly active enzyme. Phosphorylation of this loop alters its structure such that the Mg-ATP pocket becomes more accessible, thus increasing kinase activity potential [11]. The structure of Lyn’s kinase domain has been solved in an active but unphosphorylated apo form as well as in complex with several inhibitors, revealing the molecular basis for its inhibition by these drugs [12]. *In vitro* analysis indicates that the Y397 phosphorylation status potentiates the greatest activity regulation [13,14], however, *in vivo*, this may be substantially modulated by the C-terminal motif and/or SH2/SH3 domain interactions. Indeed, the activation/inactivation regulatory cycle of Lyn is further controlled by its association with different scaffold/adaptors that are restricted to different subcellular compartments, such as Cbp/PAG1, which predominantly resides within lipid rafts [15-18]. In the inactivation cycle of Lyn, phosphatases such as SHP-1 and SHP-2, which are themselves attracted to molecules phosphorylated by Lyn, are known to dephosphorylate the pY397 activation loop site, and are thus important in down-regulating this emzyme [19].

**Figure 2:**
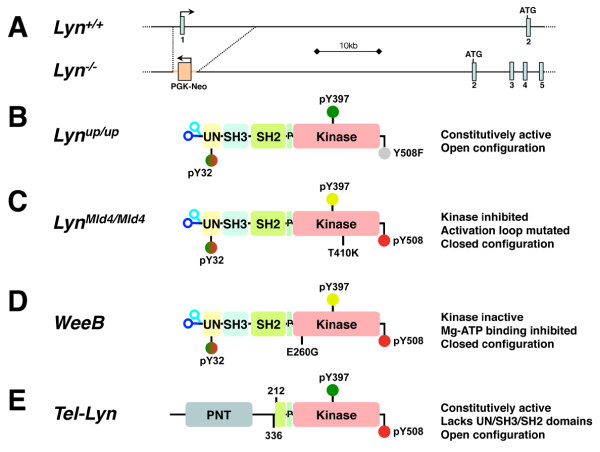
**Mutations of Lyn in genetically engineered mice.A**) Deletion of exon 1 in *Lyn*^*−/−*^ mice. Schematic of the genomic region of *Lyn* highlighting the generation of *Lyn*^*−/−*^ mice through the replacement of exon one and surrounding sequences with a PGK-Neo cassette, transcribed in the opposite orientation to the *Lyn* gene. **B**) *Lyn*^*up/up*^ mice contain a point mutation of the C-terminal tyrosine, generating a phenylalanine (Y508F) that is unable to be phosphorylated. **C**) *Lyn*^*Mld4/Mld4*^ mice contain a point mutation of a threonine at the end of the activation look, to a lysine (T410K), which inhibits the activity of the enzyme. **D**) *WeeB* mice contain a point mutation in the glycine loop, a glutamic acid is changed to a glycine (E260G), inhibiting binding of Mg-ATP, resulting in an inactive enzyme. **E**) The Tel-Lyn fusion juxtaposes the PNT domain of Tel (ETV6) with a truncated Lyn lacking its regulatory UN/SH3/SH2 domains, generating a constitutively active kinase fusion. Domains and motifs of Lyn are as described in Figure 1.

The involvement of Src family kinases including Lyn in various signaling cascades is gradually being elucidated (reviewed in [3]). While Lyn was originally identified as a hematopoietic specific kinase it is expressed in many tissues and is involved in the transmission of signals from a number of receptors such as B-cell receptor [20,21], GM-CSF-receptor [22], FcϵR1 [23], Epo-receptor [24-26], and c-kit [27], among others (reviewed in [28,29]), as well as integrins [30]. Lyn phosphorylates a number of signaling molecules, including the immunoreceptor tyrosine-based inhibitory/activation motifs (ITIM/ITAM) on PIR-B/SIRPα and FcRγ [22,31], as well as PI-3 kinase, FAK, ras-GAP, PLCγ1/2, HS1 [32], Cbp/PAG1 [15,16], STAT5 [33] and MAP kinase. *Lyn*^*−/−*^ mice (Figure 2A) are viable but display defects in their immune system [20], myeloid lineage [34], erythroid compartment [26,35], and have neuronal [36] and prostate tissue deficiencies [37]. Mice expressing a constitutively active Lyn (*Lyn*^*up/up*^, Figure 2B) also develop abnormalities of the myeloid and lymphoid systems [21,34], and our preliminary work illustrates they have erythroid abnormalities (unpublished observations). Further investigation is required to delineate any alterations to other tissues/organs in these hyperactive Lyn expressing mice. Intriguingly, *Lyn*^*up/up*^ mice do not appear more prone to developing any neoplasia, while *Lyn*^*−/−*^ animals have an age-dependent accumulation of disseminated macrophage tumors [34]. While much emphasis is placed on the kinase activity of Lyn, and indeed other SFK/tyrosine kinases, as providing the paramount down-stream signaling capacity of this enzyme, the recent identification of major immune cell signaling differences in mice carrying kinase dead alleles of *Lyn* (*Lyn*^*Mld4/Mld4*^, [38], *WeeB*[39], Figure 2C,D) compared to those that have *Lyn* deleted (*Lyn*^*−/−*^), implies there are important scaffolding functions for this molecule in addition to its enzymatic activity.

In addition to the normal function and crucial role of Lyn in B-cell receptor signaling (reviewed in [31]), as well as several growth/cytokine receptor signaling systems [29], it has also been noted to function in numerous mature as well as progenitor blood cells. While Lyn has important functions in Epo-receptor signaling in erythroid progenitors [26,35,40], it is also the most abundant SFK in mature erythrocytes [41] and phosphorylates important cell shape controllers/ion transporters in these cells e.g. Band 3, in response to physico/chemical regulators of red cell function [42,43]. Lyn is also involved in thrombopoietin(Tpo)-receptor signaling in megakaryocytes [44], as well as in mature platelets [45,46], where it functions in regulating integrin and FcRγ signaling, crucial for their function in adhesion and aggregation [47,48]. Lyn has important signaling functions in mast cells; engagement of the FcϵRI activates Lyn to regulate mast cell degranulation [49-51]. Again, in this system as in others, Lyn appears to have both positive and negative regulatory roles. A recent study highlighted a potential isoform-specific function for the two molecular species of Lyn (LynA, p56 and LynB, p53), within platelets, utilizing a background of *Lyn*^*−/−*^ cells within which the specific isoforms of Lyn were then expressed either individually of in combination [52]. Not only did LynA and LynB have differing signaling capacities, but they also displayed differential association with effector molecules. Indeed to restore the wild-type signaling, both LynA and LynB isoforms needed to be co-expressed, either isoform alone producing abnormal responses [52]. Extending these isoform specific studies to the other cell systems that involve Lyn will be of great interest, and may help delineate the molecular mechanisms behind the duplicitous nature of this enzyme.

It is clear that Lyn has important functions in numerous hematopoietic cell types, from early stem/progenitors (signaling via the stem cell factor receptor/c-kit [27]) through to multiple lineages of the lymphoid system (e.g. B-cell) and the myeloid system (e.g. macrophages, erythroid cells, platelets, mast cells, eosinophils). Interestingly, while Lyn is not expressed in T-cells, it can have a significant impact upon T-cell function through modulating signaling in cells that interact with T-cells [53]. Within these signaling pathways it is becoming apparent that Lyn has a dual role by both activating and inhibiting signaling pathways, thus it is aptly described as a signaling modulator (Figure 1B). Within the non-hematopoietic cells (e.g. prostate, colon, breast, neuronal/astrocytes) that express Lyn, its role in activating/inhibiting various signals still requires further elucidation, however, most studies so far point to it being a positive regulator [36,37,54-56].

### Immune diseases regulated by Lyn

Lyn has unique roles in B lymphocyte signaling (reviewed in [31]), mediating positive and inhibitory signals, as highlighted through the B-cell phenotypes when *Lyn* is deleted (*Lyn*^*−/−*^), inactivated (*Lyn*^*Mld4/Mld4*^*WeeB*), and hyper-activated (*Lyn*^*up/up*^) in the whole animal [20,34,38,39]. At the signaling level there are the expected major differences between *Lyn*^*−/−*^ and *Lyn*^*up/up*^ mice, i.e. reduced phosphorylation with *Lyn*-deficiency and hyper-phosphorylation with constitutively active Lyn. Dysregulation of Lyn results in antibody-mediated autoimmune disease, leading to speculation that Lyn may be a key component in such clinically similar diseases, such as systemic lupus erythematosus (SLE). While there is limited evidence of a direct involvement of Lyn in SLE [57,58], the physiological similarities between dysregulated Lyn mice and autoimmune diseases may make these animals useful models of such human diseases. It is very interesting to note that mice with defective Lyn kinase activity (*Lyn*^*Mld4/Mld4*^*WeeB*), but still expressing Lyn protein have a less severe immune dysfunction phenotype than those with Lyn fully-ablated [38,39]. It is also becoming apparent that immune diseases that are thought to be mediated by cells that don’t express Lyn, i.e. T-cells in asthma, can also be modulated by dysregulation of Lyn [53]. Inhibiting Lyn kinase activity could inhibit airway eosinophilia, as Lyn has important functions in IL-5 receptor signaling [59], in which it is associated with asthma [60]. However, the cellular changes of *Lyn*^*−/−*^ mice would suggest they would be more prone to asthma. Indeed, when *Lyn*-deficient animals are challenged with an asthma-induction model, they develop a persistent and more severe form of the disease than control mice [53]. This effect may be due to the lack of inhibitory Lyn-mediated signaling in the many non-T-cell immune regulators and mediators, i.e. B-cells, macrophages, eosinophils, neutrophils, dentritic cells and mast cells (reviewed in [22,23]), in these *Lyn*^*−/−*^ mice, resulting in an inability to negatively regulate Th2 immune responses. Further, the enhanced Th2 response in *Lyn*^*−/−*^ mice also appears to be contributed to by the lack of Lyn signals through the FcϵRI in basophils [61]. With Lyn also being intimately involved in FcϵRI signaling within mast cells, a potential to regulate mast cell inflammation associated with allergic reactions also exists (reviewed in [23]). Interestingly, while *Lyn*^*−/−*^ B cells can be autoreactive, the immune disease that they cause is strongly linked to the IL-6-dependent inflammatory environment that they induce [62]. Additionally, hyperactive myeloid cells produce BAFF (B lymphocyte stimulator) that activates B-cells, but also activates T-cells to release IFNγ, creating an inflammatory loop that exacerbates the autoimmunity of *Lyn*^*−/−*^ animals [63].

There is some interesting data suggesting that the subcellular localization of Lyn is crucial for its normal functioning [64-66]. Lyn can be cleaved by caspase 3 and 7 within its N-terminal unique region, resulting in removal of the myristoylation and single palmitoylation sites, resulting in the cleaved form localizing exclusively to the cytosolic compartment, while full-length Lyn can be found predominately in the plasma membrane (often within lipid rafts) as well as the cytosol [64,66]. The production of this cleaved form of Lyn occurred in B-cells in response to induction of apoptosis, such as via BCR ligation [66] and functions as a suppressor of apoptosis [64]. Intriguingly, when this cleaved form of Lyn is ubiquitously over-expressed in transgenic mice [65], STAT3 is activated and NFκB inhibited, and it induces a skin inflammatory syndrome, very similar to that seen in psoriasis. Indeed in skin biopsies of psoriasis sufferers the cleaved form of Lyn could be detected. This phenotype was dependent upon the presence of a TNFα/TNF-receptor axis, and was ameliorated in *Rag1*-deficient mice highlighting the involvement of a T-cell response in the observed pathogenesis. Consequently, not only is the activity status of Lyn important for immune cell function, but also its sub-cellular localization.

### Leukaemia/Lymphoma and Lyn

The level and/or activity of tyrosine kinases including members of the Src family kinases (SFK) are often elevated in human neoplastic cells and their activity can correlate with disease severity/metastatic potential [67-69]. Recent advances in the development of small molecule inhibitors of tyrosine kinases has resulted in great success in treating particular neoplasms and the therapeutic advantages of these reagents is illustrated by the enormous success of Imatinib mesylate (Gleevec, STI571) and Dasatinib for the treatment of CML [70], and the recent release of Tykerb (lapatinib ditosylate) [71] and Sutent (Sunitinib) [72] for the treatment of solid tumours such as breast, lung and renal cancer.

While *in vivo* manipulation of Lyn through knockout (*Lyn*^*−/−*^), inactivation (*Lyn*^*Mld4/Mld4*^, [38]), and hyper-activation (*Lyn*^*up/up*^) mouse models have not provided strong evidence for this kinase having a primary role in initiating neoplasia, substantial evidence does exist implicating it in regulating cancer/leukaemia cell biology [37,55,73-76]. These studies are suggestive that Lyn can be utilized in an oncogene addiction role in these neoplastic cells, subsequent to transformation initiation events involving other molecules. Further, it will be useful to undertake crosses of *Lyn*^*−/−*^ and *Lyn*^*up/up*^ mice with other regulators of oncogensis to delineate pathway interactions. A recent study found that combining a lack of Lyn with a lack of phospholipase beta 3 (PLCβ3) causes a chronic myelomonocytic leukaemia through reduced SHP-1 activity resulting in enhanced STAT5 phosphorylation [77].

Evidence is mounting that strongly implicates an important role for Lyn in several types of leukaemia and lymphoma, especially chronic myeloid leukaemia (CML) [76], acute myeloid leukaemia (AML) [75,78], chronic lymphocytic leukaemia (CLL) [79], B-cell acute lymphoblastic leukaemia (B-ALL) [74], B-cell chronic lymphocytic leukaemia (B-CLL) [79], and B-Non Hodgkin’s lymphomas (B-NHL) [80]. Recently, Lyn has also been identified as a fusion in myelo-proliferative disorders [81].

An important role for Lyn in both chronic and acute myeloid leukaemic cells has been suggested by several studies [75,76,82-84]. In an analysis of primary AML cells, 76% had elevated Lyn kinase activity, while none had constitutive JAK2 activation; moreover, inhibition of Lyn activity (using genetic and small molecule inhibitors) in AML cell lines substantially decreased cell growth [75]. Recent studies have also confirmed the common activation of Lyn in primary AML, and its critical role in maintaining proliferation and anti-apoptotic pathways in these cells [78]. Further, Lyn is a signaling component of the Fms-like tyrosine kinase 3/internal tandem duplication (FLT3/ITD)-specific pathway linking FLT3/ITD to STAT5. FLT3/ITD is the most common mutation in human adult AML and Lyn binds with high affinity to this mutated receptor. Down-regulation of Lyn/Src family kinases in these AML cells by siRNA or small molecule inhibitors substantially ameliorated their growth *in vitro* and tumor establishment and growth *in vivo*, as well as phosphorylation of important down-stream FLT3/IDT mediators such as STAT5 [85]. One of the standard treatments for AML is through differentiation induction by all-trans-retinoic acid (ATRA), and recent data has shown a potentially important modulation of this pathway by Lyn [86]. Here, Lyn inhibitors, such as Dasatinib, enhanced the effectiveness of ATRA through a positive feedback loop involving a scaffolding protein (KSR1) that complexes regulators of the MAP kinase pathway with Lyn, illustrating the potential for using Lyn/SFK inhibitors as adjunct therapies in combination with other anti-AML effectors [86].

A number of reports have illustrated that while the BCR-Abl fusion protein is the initiating molecule for CML, there is a crucial down-stream role for Lyn in BCR-Abl induced leukemogenesis [82,83]. Not only does Lyn bind BCR-Abl and is activated by it [84], but Lyn can also phosphorylate BCR-Abl and modulate its ability to transform cells [87]. There are other direct links between Lyn and BCR-Abl signaling pathways as Lyn phosphorylates the Y177 motif of BCR-Abl [88], resulting in recruitment of the adaptor Gab2, a principle activator of the PI-3 kinase pathway, both of which are essential for BCR-Abl oncogenesis [89]. Lyn is also involved in other signaling pathways in CML cells, including through BCR-Abl activation of JAK2 that then activates Lyn by preventing SHP-1 from turning off Lyn activity [90]. Interestingly, when the CML cell line K562 is selected for Imatinib resistance, the resultant cell line (K562R) has elevated Lyn levels and kinase activity, as apposed to BCR-Abl mutations; their sensitivity to this drug is regained upon down-regulation of Lyn [76]. Significantly, in primary CML cells from patients that have acquired Imatinib resistance, elevated Lyn levels also appear commensurate with the development of this drug resistance [76]. A study of drug-resistant blast crisis BCR-Abl (+) leukaemic cells showed that Lyn is crucial for their survival. Ablation of Lyn from these cells resulted in the induction of apoptosis, while normal CD34+ stem cells were not affected by Lyn ablation [91]. Detailed phosphoproteomic analysis of Bcr-Abl transformed cells [92] has revealed significant cross talk between Bcr-Abl and the negative feedback loops that control Lyn signaling via Cbp/PAG1. The protein Cbp/PAG1 [15,17,18] is a scaffold molecule involved in recruiting both the inhibitory kinase Csk, which inturn also recruits the inhibitory phosphatase PTPN18 [93], as well as E3 ubiquitin ligase SOCS1 to active Lyn, via facilitating the enzymatic inactivation of Lyn (through Csk phosphorylation of the C-terminal tyrosine of Lyn, and PTPN18 dephosphorylation of the activation loop motif) as well as degradation of Lyn via the proteasome through its poly-ubiquitination mediated by SOCS1. In these CML cells the Bcr-Abl kinase overpowers the negative feedback loops initiated by its activation of Lyn through activation of the phosphatase Shp2 that is able to dephosphorylate Cbp/PAG1 thus mitigating its ability to turn off the Lyn signals [92]. It is also very interesting to note that the second generation (T315I non-effective, e.g. Dasatinib [94] and Bafetinib [95]) and third generation (T315I effective, e.g. Ponatinib [96]) anti-CML drugs, predominantly developed to combat the different point mutations in BCR-Abl that are the more common causes of Imatinib resistance, are also potent and effective inhibitors of Lyn [12]. It will be interesting to see if these inhibitors that have relatively few side-effects are useful chemotherapeutic agents for other leukaemias/lymphomas or even solid tumors that are shown to utilize Lyn for maintaining their neoplastic state, or in other diseases that appear to involve Lyn, e.g. autoimmune diseases.

Interestingly, in B-Non Hodgkin’s lymphomas (B-NHL) there appears to be a Lyn/Cbp/STAT3 signaling complex, not present in ALK^+^ T lymphoma or Hodgkin-derived lymphoma cells, that doesn’t contain the Lyn inactivating Csk kinase and promotes survival signals in these lymphomas. When this signaling complex was inhibited or down-regulated, these lymphoma cells had substantially reduced survival [80]. This study suggests that neoplastic cells may hijack Lyn complex mediators, i.e. Cbp/PAG1, that are normally involved in turning off Lyn signals, and transforming them from these inhibitory regulators [15-18] to positive signaling mediators.

B-cell chronic lymphocytic leukaemia (B-CLL) cells contain anomalous Lyn levels, much higher than those seen in normal B-cells (which are a major cell type for natural Lyn expression). In B-CLL cells Lyn is present throughout the cytoplasm and not just localized to the plasma membrane as in normal B-cells. Further, they have substantial basal Lyn kinase activity that is un-responsive to IgM stimulation, unlike that seen in non-malignant cells. Small molecule Lyn inhibitors were effective at inducing apoptosis in these B-CLL cells suggesting that Lyn contributes to negating the apoptosis pathway in this form of leukaemia, and suggests altered localization of Lyn can contribute to its involvement in oncogensis [79]. Interestingly, in B-CLL cells overexpressing the phosphatase PTPN22, their acquired inhibition of antigen-induced apoptosis and positive regulation of an anti-apoptotic Akt pathway, is due to a selective uncoupling of the Akt pathway that Lyn regulates down-stream of the B-cell receptor [97]. Here PTPN22 dephosphorylates the activation loop of Lyn, turning off its kinase activity, and consequently its pro-apoptotic pathways down-stream of the B-cell receptor [97]. Taken together, these studies suggest that its not just the level of Lyn activity that is important but also its localization and interaction with regulators that can influence weather or not it functions as a positive effector or negative regulator in B-cells/B-cell leukaemia.

The potential for Lyn’s kinase activity to have a direct capacity to promote a myeoproliferative/leukaemic phenotype was illustrated by studies screening a cDNA library fused to Tel (ETV6), a common fusion partner in AML [98]. Here Tel-Lyn was identified in nearly half of all the transforming initiating independent fusion events. Subsequently, Tel-Lyn fusions (Figure 2E) have now been identified in primary myelofibrosis patients with blastic transformation [99]. This fusion between Tel and the kinase domain of Lyn directly activates STAT5, independent of JAK2, to cause neoplastic transformation and myelofibrosis, which was substantially dependent upon STAT5, i.e. removal of STAT5 essentially prevented the Tel-Lyn fusion from inducing myelo-proliferation [81]. It is interestingly to speculate that the patient with the Tel-Lyn fusion could have benefited from treatment with second or third generation CML drugs (e.g. Dasatinib), which are highly potent Lyn kinase inhibitors, instead of the Immatinib therapy they received prior to succumbing to their disease.

These studies show that Lyn, while rarely a primary causative agent in leukaemia/lymphoma, is nonetheless often intimately involved in the oncogenic signaling cascades within these neoplastic cells. Further, that several cases illustrate that altering Lyn complexes and localization appear important for Lyn to play an important oncogenic role. While altering just its kinase activity without altering its signaling complexes/localization may have minimal oncogenic consequences. Consequently, the development and use of anti-Lyn/anti-Lyn-pathway therapies has potential usefulness in the treatment of certain types of leukaemia/lymphoma.

### Involvement of Lyn in solid tumors

Considerable evidence implicates tyrosine kinases in the development of many types of solid cancer as well as leukaemia via their involvement in numerous growth factor signaling cascades (reviewed in [100]). Ever since the discovery of the transforming capacity of the Src family of kinases, indeed Src was the first identified oncogene, causing sarcomas in chickens [101], substantial effort has gone into determining if this family of genes are mutated in human cancers. While only a few instances have found them to be genetically altered in solid cancer [102], their activity/levels, as illustrated recently [103], are commonly altered. Lyn has also been specifically implicated in solid cancers, namely prostate cancer [37], colon cancer [54], Ewing’s sarcoma [104], glioblastoma [56], and breast cancer [105]. Indeed, recent proteomic analysis of 130 tumor lines showed that Lyn, as well as Src and Lck are some of the most consistently activated tyrosine kinases in many types of cancer cells [103].

Expression of Lyn has been detected in colorectal tumours that are metastatic, but not at earlier stages of cancer development or in normal tissue [106]. Colon carcinoma cells utilize Lyn downstream of a variant form of the cell surface CD44 molecule in the activation of the Akt anti-apoptotic pathway, and drug-resistant cells show elevated Lyn kinase activity [54]. Further, this pathway also appears to regulate the directional migration of these cells [107]. Surprisingly, Lyn is expressed at high levels in most prostate cancer cell lines and primary prostate tissue [37,108], and *Lyn*^*−/−*^ mice have significantly smaller prostates with fewer proliferating cells than wild-type animals [37]. Lyn also regulates signaling mechanisms in prostate cancer cells that influence cell migration [108], and primary prostate cancer tissues have elevated Lyn levels. Significantly, inhibition of Lyn in prostate cell lines using a peptide designed to act as a competitive inhibitor of the substrate binding site of the kinase domain, resulted in reduced proliferation not only *in vitro* but also in mice carrying prostatic cancer xenografts [37]. The level of inhibition correlated with the level of Lyn expression in these prostate cell lines, supporting the proposed mechanism of action of this peptide inhibitor [37]. Further, targeting Lyn using the small molecule inhibitor Dasatinib substantially reduces lymph node metastasis of prostatic cancer cells as well as the growth of the primary xenografted tumor [109]. Intriguing data looking at the ability of different SFKs to initiate prostate epithelia transformation showed Src and to a lesser extent Fyn, but not Lyn, have this capacity when expressed in their hyper-active forms, partially dependent upon their different N-terminal lipid modifications [110]. Potentially, these studies lend support to the hypothesis that transformed cells may utilize Lyn in the maintenance of their phenotype, making it a legitimate therapeutic target, but that Lyn itself is not a primary initiator of transformation.

In Ewing’s sarcoma, a poorly differentiated bone/soft tissue cancer that is characterized as sharing a common translocation generating an EWS-ETS fusion, Lyn also appears as a potential therapeutic target [104]. Using RNAi or small molecule inhibitors to down-regulate Lyn, the growth and metastatic capacity of these Ewing’s sarcoma lines was substantially inhibited both *in vitro* and *in vivo*. It also appeared that the EWS-ETS fusion, a transcription factor, up-regulated *Lyn* gene expression [104]. In a survey of primary glioblastomas, Lyn was found to be the predominant active SFK, compared to normal brain, or indeed other types of brain tumors [56], supporting previous work showing Lyn promotes PDGF-mediated migration of glioblastoma cells [111].

Lyn pathways are also involved in signaling mechanisms within breast cancer cell lines [105], but it’s expression is apparently not high or wide-spread in breast cancers. Undertaking more detailed analysis of Lyn expression in primary breast cancers and cells lines derived from these tumors has revealed important functions for Lyn in this type of cancer [55,73]. Stratifying breast cancer lines by epithelial/mesenchymal morphology identified Lyn as associating with a mesenchymal or rather a “basal” type breast cancer morphology, which also correlated with reduced overall survival [73]. Basal breast cancers are known to be more aggressive than their luminalA/B, normal-like and HER2 counterparts [112]. Knocking down Lyn in basal type breast cancer cell lines inhibited their migration and invasion, but not proliferation, *in vitro*. In addition, using Dasatinib at concentrations known to inhibit Lyn, also repressed the invasive capacity of these cells, but didn’t’ affect their proliferation. A similar association of Lyn with basal breast cancer was also uncovered though phospho-tyrosine proteomic profiling of breast cancers [55]. Again, in the basal type cells elevated phosphorylation of Lyn, as well as Met, EphA2, EGFR, FAK and p130Cas were noted. Inhibiting or down-regulating these different active kinases in these cells all showed significant effects on their biology, including proliferation, migration and invasion. Combining inhibitors of these different kinases had a greater effect, in particular a receptor kinase, i.e. EGFR, and a cytoplasmic kinase, i.e. Lyn. SFK signaling networks, in particular Lyn, appeared to be central to the basal cell phenotype, intersecting numerous pathways involving the other activated tyrosine kinases in these cells [41]. Interestingly, Lyn is also present in luminal progenitors, thought to be the cells-of-origin for basal breast cancer, supporting speculation that abnormal Lyn signaling helps drive the basal cancer cell phenotype.

Thus several lines of evidence point to a significant involvement of Lyn in solid tumor development, as well as leukaemias. Further, solely deleting or hyper-activating Lyn on its own has little direct influence upon the prevalence of leukaemia or cancer [20,34], but as this enzyme is important for particular neoplasms (reviewed above), it must therefor be concluded that a dysregulation of pathways/substrates/regulators/adaptors, intersected by Lyn, rather than just Lyn itself, is what potentiates these particular cancers and leukaemias.

## Conclusions

Numerous cell lineages of the hematopoietic system (with the notable exception of T-cells), as well as progenitor cells, utilize Lyn in complex signaling pathways, often through regulating receptor and/or integrin initiated networks. It’s critical role in B-cell receptor signaling has highlighted its modulating capacity via activating as well as inhibiting down-stream pathways. Phosphorylation and protein-protein interactions regulate Lyn activity, and new data suggests the two isoforms of Lyn, which differ in the presence/absence of a pY motif (pY32), may have different biological functions, potentially explaining its duality in activating and inhibiting signaling. Lyn’s regulation of immune cell function suggests it may be important for immune diseases; indeed dysregulation of Lyn can lead to autoimmune diseases in mice, reminiscent of SLE, asthma and psoriasis. While Lyn rarely appears as an initiator of leukaemogenesis, mounting evidence implicates it in playing a role in maintaining the leukemic phenotype in a variety of liquid cancers, including AML, CML, B-ALL and B-CLL. In addition to being a hematopoietic kinase, Lyn is also expressed in other tissues, and has a recognized oncogenic role in solid cancers of these organs, including prostate, basal breast, and colon cancer, as well as Ewing’s sarcoma and glioblastoma. With several anti-cancer drugs being potent Lyn inhibitors, it is going to be important to address the role that Lyn and its pathways play in these different neoplasms.

## Abbreviations

SFK, Src Family Kinases; AML, Acute Myeloid Leukaemia; CML, Chronic Myeloid Leukaemia; CLL, Chronic Lymphocytic Leukaemia; B-CLL, B-Cell Chronic Lymphocytic Leukaemia; B-NHL, B-Non Hodgkin’s lymphoma; SH, Src Homology; SLE, Systemic Lupus Erythematosus; BCR, B-Cell Receptor.

## Competing interests

The author declares that he has no competing interests.

## Authors’ contributions

EI organized, wrote and edited the manuscript, as well as designed the figures.

## Authors’ information

EI heads the Cell Signalling Group, WAIMR, Perth, WA 6000, Australia. Additional information on the research undertaken by the author can be accessed at http://www.waimr.uwa.edu.au/team/eingley.html.
